# *Escherichia coli,* but Not *Staphylococcus aureus,* Functions as a Chelating Agent That Exhibits Antifungal Activity against the Pathogenic Yeast *Candida albicans*

**DOI:** 10.3390/jof9030286

**Published:** 2023-02-22

**Authors:** Swagata Bose, Durg Vijai Singh, Tapan Kumar Adhya, Narottam Acharya

**Affiliations:** 1 Department of Infectious Disease Biology, Institute of Life Sciences, Bhubaneswar 751023, India; 2 KIIT School of Biotechnology, Bhubaneswar 751021, India; 3 Department of Biotechnology, School of Earth, Biological and Environmental Sciences, Central University of South Bihar, Gaya 824236, India

**Keywords:** candidiasis, morphogenesis, nutritional immunity, peptidoglycan, biofilm, pathogenesis, quorum sensing, divalent metals

## Abstract

Humans are colonized by diverse populations of microbes. Infections by *Candida albicans*, an opportunistic fungal pathogen*,* are a result of imbalances in the gut microbial ecosystem and are due to the suppressed immunity of the host. Here, we explored the potential effects of the polymicrobial interactions of *C. albicans* with *Staphylococcus aureus*, a Gram-positive bacterium, and *Escherichia coli*, a Gram-negative bacterium, in dual and triple in vitro culture systems on their respective growth, morphology, and biofilms. We found that *S. aureus* promoted the fungal growth and hyphal transition of *C. albicans* through cell-to-cell contacts; contrarily, both the cell and cell-free culture filtrate of *E. coli* inhibited fungal growth. A yet to be identified secretory metabolite of *E. coli* functionally mimicked EDTA and EGTA to exhibit antifungal activity. These findings suggested that *E. coli*, but not *S. aureus*, functions as a chelating agent and that *E. coli* plays a dominant role in regulating excessive growth and, potentially, the commensalism of *C. albicans*. Using animal models of systemic candidiasis, we found that the *E. coli* cell-free filtrate suppressed the virulence of *C. albicans*. In general, this study unraveled a significant antimicrobial activity and a potential role in the nutritional immunity of *E. coli*, and further determining the underlying processes behind the *E. coli–C. albicans* interaction could provide critical information in understanding the pathogenicity of *C. albicans*.

## 1. Introduction

The human microbiota is a community of microorganisms that reside on or within the human organs and biofluids. These microbes could be commensal, symbiotic, or pathogenic in nature [1,2]. These are the species of bacteria, archaea, fungi, protists, and viruses. Since bacteria by far are the most abundant, they could be highly influential among the microbes in regulating the microbiota-induced host physiology and development. It is well accepted that, while microbial homeostasis results in a net benefit to the host, dysbiosis in this complex community is associated with numerous negative health outcomes. Thus, a complex polymicrobial association that naturally persists in humans can be either beneficial or detrimental to the host [3]. Candidiasis is a fungal disease caused by the *Candida* sp. in humans, and the microbial dysbiosis and compromised immunity of the host are the two main causative factors of candidiasis [4]. It carries about a 40–60% mortality rate [5,6]. It represents the fourth and sixth most prevalent nosocomial infections in the United States and Europe, respectively. According to the CDC estimation, about 10 per every 100,000 people are affected by candidiasis every year. Nearly 25,000 such cases appear nationwide each year, and it is expected to affect nearly 500,000 people each year worldwide. Unfortunately, due to poor diagnoses, the unavailability of approved vaccines, and limited antifungal drugs, the mortality rate due to fungal infections is as high as that caused by tuberculosis or HIV [5]. Most of these infections are caused by *Candida albicans. C. albicans* is a polymorphic fungus, and its morphological switch from yeast to a hyphal form is one of its major virulence determinants [7]. Yeast cells are a benign trait; however, they can disseminate into the bloodstream easily, whereas hyphae are invasive and can escape phagocytosis by the innate immune cells. While both forms of *C. albicans* are required for virulence, growing evidence suggests that the yeast-shaped *C. albicans* is the commensal state and is usually found in the oral cavity, gut, and genitourinary tract of healthy individuals [3,8]. Recently, by using animal models and dietary *C. albicans*, we showed that *C. albicans* maintains a mutualistic relationship with the murine host and regulates the microbiome dynamics in the gut, the food intake, and the metabolism [4]. Since *C. albicans* coexists in harmony with other resident colonizers, the bacterial–fungal interaction could determine the commensal-to-pathogenic transition of *C. albicans*. Several reports have suggested that the excessive use of broad-spectrum antibiotics causes dysbiosis, which facilitates *C. albicans* colonization and its invasive growth, as the killing of bacteria creates enough empty sites on the host epithelial tissues for fungal adhesion and reduces competition for nutrients and other resources [9,10,11]. However, studies have also found that the depletion of the bacterial population alone in the colonized sites is not enough to promote the invasive growth of *C. albicans*; additionally, it requires the immunosuppression of the host and damage to the mucosal layer [12,13]. Since severe *C. albicans* infections are relatively rare in healthy individuals and occur mostly in immune-suppressed individuals and because our observation that a high-fat diet that induced the excessive colonization of *C. albicans* did not cause any infections in mice, the function of the host’s immune system seems to be equally as critical as microbial homeostasis in the prevention of *C. albicans* infections [4,5]. Thus, a tripartite interaction between *C. albicans,* neighboring bacteria, and the immunity of the host is most likely the key to the onset of candidiasis.

There are enough studies to suggest that the microorganisms in a community can alter each other’s growth and antimicrobial susceptibility [3,14,15]. *Staphylococcus aureus* mucosal infections and *Pseudomonas aeruginosa* respiratory tract infections are promoted by *C. albicans* colonization and their interactions with the fungal hyphae [16,17,18]. However*, P. aeruginosa* induces fungal death through the production of phenazine derivatives [19]. Similarly, lactic acid bacteria compete for adhesion sites and secrete inhibitory substances to control the *C. albicans* invasion and disease [20]. Mutualism between *C. albicans* and *Streptococcus mutans* has also been seen. *C. albicans* utilizes the glucose produced by *S. mutans* after it metabolizes sucrose, and this degradation of glucose creates a conducive acidic environment for both the microbes to grow parallelly [21]. *C. albicans* and *E. coli* exhibit a cooperative interaction wherein *E. coli* enhances the adhesion of *C. albicans* to the bladder mucosa and where it enhances the occurrence of urinary tract fungal infections [22]. Contrary to this study, a recent report suggested that *E. coli* inhibits *C. albicans* growth in vitro [23]. Undoubtedly, both the commensal and pathogenic status of *C. albicans* seems to be influenced by its direct and indirect interactions with bacteria. In comparison to their pathogenic facets, very little is known about the complex interactions and signaling events that occur between the microbes that restrict *C. albicans* to growing predominantly in a commensal state. In this study, we revisited the polymicrobial interaction of *C. albicans* with *S. aureus*, a Gram-positive bacterium, and *E. coli*, a Gram-negative bacterium, in dual and triple in vitro culture systems. Further, we showed that *E. coli*, but not *S. aureus*, functions as a chelating agent that exhibits antifungal activity to suppress the growth and virulence of *C. albicans* using an animal model of systemic candidiasis. Thus, *E. coli* could play a predominant role in the gut in suppressing the commensal-to-pathogenic transition of *C. albicans.*

## 2. Results

### 2.1. Differential Effects of S. aureus and E. coli on the Growth of C. albicans in the Coculture Condition

**Figure 1:**
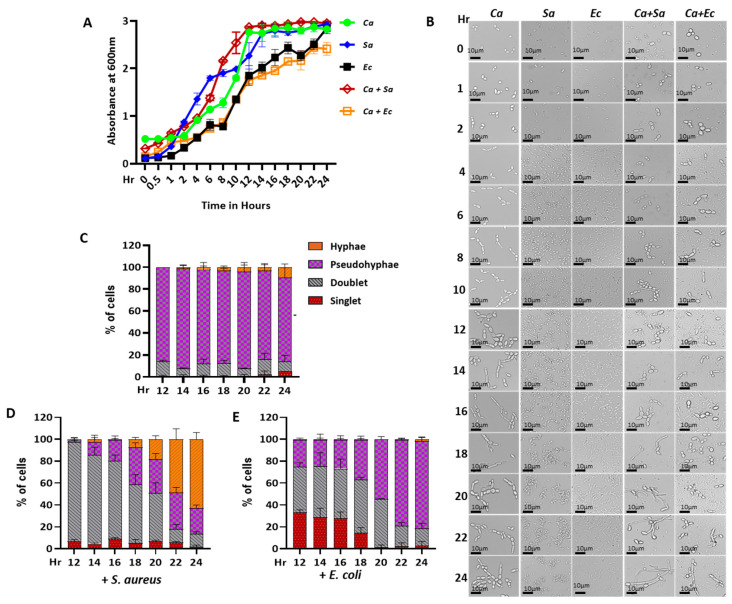
Microbial growth and morphology analyses. Cells of *C. albicans* (*Ca*), *S. aureus* (*Sa*), and *E. coli* (*Ec*) were inoculated in YPD media as pure and mixed cultures and were allowed to grow at 37 °C, and their absorbance at OD_600nm_ was monitored for 24 h in single- (*Ca* is represents by a green line, *Sa* is represented by a blue line, and *Ec* is represented by a black line) and dual-culture (*Ca* + *Sa* is represented by a brown line; *Ca* + *Ec* is represented by an orange line) conditions. The plot was the average of two sets with three independent experiments (**A**). Cells at mentioned time points were imaged under a 40× Leica DM500 microscope (**B**)**.** The percentage of singlet, doublet, pseudo-hyphae, and hyphae in *C. albicans* cells as color-coded were estimated in the fungal monoculture (**C**), or in the presence of *S. aureus* (**D**), and *E. coli* raw cultures (**E**).

In order to explore the cell-to-cell communication between *C. albicans* and the bacterial strains, we performed growth curve analyses and determined the efficiency of the colony-forming units (CFU) of each microbe in coculture conditions. For these analyses, we selected two differentially Gram-stained bacteria, *E. coli* (MG1655) and methicillin-resistant *S. aureus (*ATCC43300), as their coexistence with *C. albicans* (SC5314) in the human gut and the other organs is well established. Shape- and size-wise, both these bacteria are also different from *C. albicans* cells, which helped in identifying the microbes in mixed-culture conditions. Additionally, the *C. albicans* colonies could be verified based on their susceptibility to amphotericin B (AmpB), while the *E. coli* and *S. aureus* strains could be selectively selected through the addition of ampicillin (AMP) and chloramphenicol (CHL), respectively. Among several media tested, the nutrient-rich YPD medium was found to be appropriate for both mono- and cocultures, where all three microbes grew efficiently at 37 °C in 200 RPM shaking conditions. Our growth curve analyses over a period of 24 h revealed almost similar kinetics of growth in the mono- and cocultures of *C. albicans* and *S. aureus* in liquid YPD media (Figure 1A). Although the growth rates of the *E. coli* pure culture and the coculture of *E. coli* and *C. albicans* were very similar, these cultures grew relatively slower than other three cultures (the black and orange lines). To confirm the presence of both the microbes in the cocultures, we observed their morphologies at different time points. Both *S. aureus* (spherical-shaped) and *E. coli* (rod-shaped) cells were found in the monoculture as well as in the coculture, and they maintained their native morphologies throughout the growth period. In the late stationary phase (18–24 h), *S. aureus* showed a minor aggregation. The majority of the *C. albicans* cells (~80%) were pseudohyphal (branched) in the monoculture from the early to late stationary phase (12–24 h, Figure 1B,C). The percentage of pseudohyphal cells was drastically reduced in both cocultures. Interestingly, the presence of *S. aureus* induced the pseudohyphae-to-hyphae transition (elongated tube-like structures) of *C. albicans* (Figure 1B,D). A higher percentage of singlet and doublet fungal cells were found in the *C. albicans*–*E. coli* coculture (Figure 1B,E).

**Figure 2:**
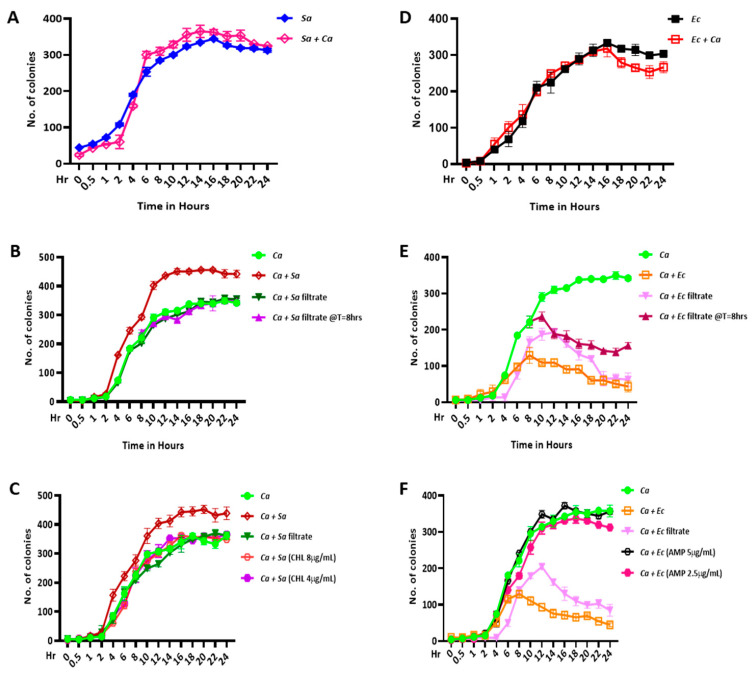
CFU analyses in mono- and dual-culture conditions. An equal number of cells (OD_600nm_ = 0.5) of *C. albicans* (*Ca*), *S. aureus* (*Sa*), and *E. coli* (*Ec*) were inoculated in YPD media as pure and mixed cultures and were allowed to grow at 37 °C, and their CFUs were monitored at mentioned time points (0–24 h). Bacterial culture filtrates were added as required instead of pure cultures. (**A**) CFU count of *Sa* pure culture and coculture with *Ca* in YPD + Amp B media. (**B**) CFU count of *Ca* pure culture and coculture with *Sa* pure culture added at T = 0 h and *Sa* culture filtrate added at T = 0 h and T = 8 h in YPD + CHL media. (**C**) CFU count of *Ca* pure culture and coculture with *Sa* culture filtrate and *Sa* pure culture without or with treatments of 8 µg/mL and 4 µg/mL concentrations of chloramphenicol and further selection in YPD + CHL media. (**D**) CFU count of *Ec* pure culture and coculture with *Ca* in YPD + Amp B media. (**E**) CFU count of *Ca* pure culture and coculture with *Ec* pure culture added at T = 0 h and *Ec* culture filtrate added at T = 0 h and T = 8 h in YPD + AMP media. (**F**) CFU count of *Ca* pure culture and coculture with *Ec* culture filtrate and *Ec* pure culture without or with treatments of 5 µg/mL and 2.5 µg/mL concentrations of ampicillin and further selection in YPD + AMP media. Mean values from three independent experiments are shown, and error bars represent the SEM.

Since the optical density measurement was insufficient for finding the impact on the microbial growth of a specific organism in a mixed culture, we sought to confirm each microbe’s effect on microbial growth by estimating the colony-forming units (CFUs) of each microbe. The addition of amphotericin B to the medium facilitated the selective growth of bacteria in the coculture condition. We noticed that the CFUs of *S. aureus* and *E. coli* were not altered significantly when their dual cultures were spread on YPD plates with amphotericin B, and the colony counts were very similar to their respective monocultures (Figure 2A,D). However, we found opposing effects of the bacterial strains on the *C. albicans* CFUs. When the *C. albicans–S. aureus* mixed culture was spread on YPD + chloramphenicol agar plates, a significant increase in the CFUs of *C. albicans* in comparison to its monoculture, which was grown for a similar duration of time, was observed (Figure 2B, the brown and green lines). Moreover, we found a similar increase in the CFUs of *C. albicans* when the *S. aureus* culture was exogenously added during the mid-log phase of *C. albicans* (after 8 h of independent growth). However, unlike in the pure culture, the fungal population in the presence of the *S. aureus* cell-free culture filtrate did not increase significantly in the monoculture (Figure 2B). In the case of the *C. albicans*–*E. coli* dual-culture condition, in contrast to *S. aureus*, *E. coli* showed an antagonistic behavior with respect to the fungal yield both in the pure culture as well as the cell-free culture filtrate forms (Figure 2E, the comparison of the green line with the orange and purple lines). A similar impact of *E. coli* on the mid-log phase culture of *C. albicans* was also observed. Our data indicated that both the bacterial cultures portrayed opposite roles in *C. albicans* growth in the coculture setup, and this also suggested that the polymicrobial interaction might be much more complex in the gut and other organs of the hosts, where several bacterial species reside and may have different impacts on fungal growth. Furthermore, to ascertain the differential effects of the *S. aureus* and *E. coli* cultures on fungal growth specific to the viable bacterial cells in the cocultures, we treated the cocultures with lethal and sublethal concentrations of CHL or AMP antibiotics, and then the CFUs of *C. albicans* were estimated. We found that, unlike the untreated cultures, both the bacterial strains, when treated with lethal and sublethal concentrations of antibiotics, had a nonsignificant effect on *C. albicans* growth (Figure 2C,F) and that the opposing effects of the bacterial strains were nullified. We observed that the methicillin-resistant *S. aureus* cells showed lethality at an 8 µg/mL CHL concentration, whereas the *E. coli* cells were abolished entirely by 5 µg/mL of AMP. These results suggested that, while *S. aureus* promoted fungal growth and hyphal transition of *C. albicans*, both the live cells and cell-free culture filtrate of *E. coli* inhibited such phenotypes of *C. albicans.* Additionally, it also suggested that cell-to-cell contact may not be necessary for *E. coli*’s influence on *C. albicans*.

### 2.2. Differential Effect of Bacteria on the Biofilm of C. albicans

**Figure 3:**
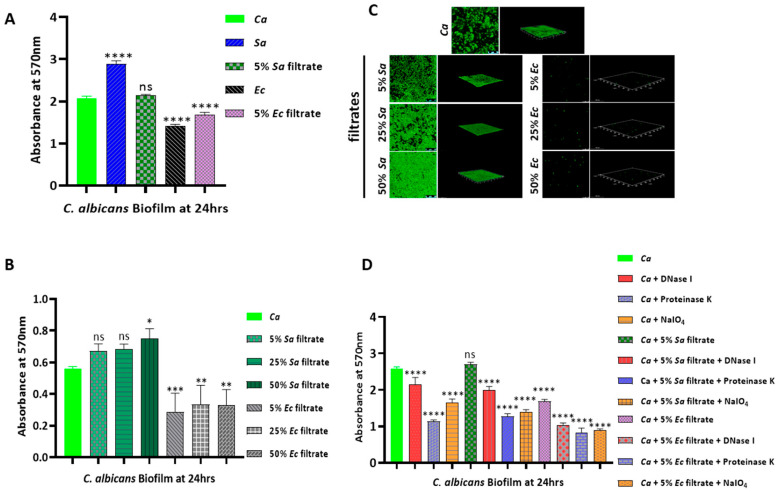
Biofilm formation and estimation through Crystal violet staining and CLSM of bacterial-filtrate-treated *C. albicans*. (**A**) Preformed biofilm of *Ca* was treated with 5% of each bacterium pure culture and their cell-free culture filtrates on a 24-well polystyrene plate for 24 h and then was estimated after crystal violet staining at 570 nm. (**B**) Preformed biofilm of *Ca* was treated with different percentages (5%, 25%, and 50%) of each bacterial culture filtrate on a 96-well polystyrene plate for 24 h and was then estimated after crystal violet staining at 570 nm. (**C**) Similarly developed biofilms were stained with 1% acridine orange in an 8-well chambered slide and were analyzed through confocal scanning microscopy. (**D**) The composition of *Ca* biofilm matrix was assessed upon treatment with DNase I, proteinase K, and NaIO_4_ on a 24-well polystyrene plate for 24 h, was developed through crystal violet staining, and had its absorbance measured at 570 nm. Graphs were plotted using GraphPad Prism 8. Mean values from three independent experiments are shown, and error bars represent the SEM. *p*-values that were * < 0.05, ** < 0.01, *** <0.001, and **** < 0.0001 were significant, and those that were > 0.05 were non-significant (ns).

A biofilm is a microbial reservoir by nature, where cell-to-cell communication is very common [24]. Since the microbes in biofilms behave differently than their planktonic cells, we thought to verify the bacterial–fungal interaction in a biofilm setup. The effects of *S. aureus* and *E. coli* on the preformed biofilm of *C. albicans* were determined through a crystal violet staining assay and through confocal laser scanning microscopy (CLSM). Our crystal violet staining suggested enhanced biofilm formation by *C. albicans* through the exogenous addition of *S. aureus* cells but not by its cell-free culture filtrate (Figure 3A). However, only after the addition of a higher volume of the *S. aureus* culture filtrate (50%) we could find a marginal increase in *C. albicans* biofilm formation (Figure 3B). On the other hand, both the pure culture of *E. coli* and its cell-free culture filtrate profoundly inhibited *C. albicans* biofilm formation (Figure 3A,B). A total of 5% of the *E. coli* culture filtrate was sufficient for inhibiting fungal biofilm on a polystyrene plate. Furthermore, our biofilm assay was validated by microscopic images (CLSM), where the *C. albicans* cell density increased due to the *S. aureus* culture filtrate, while the *E. coli* culture filtrate abolished *Candida* biofilm formation (Figure 3C). Thus, these results suggested that the bacterium that supported candidal growth also supported biofilm development and vice versa. We also checked the biofilm formation capacity of both the bacterial strains in the presence of the pure culture and the filtrate of the *C. albicans* culture. We noticed a negligible increase in *S. aureus* biofilm formation (Figure S1A), but an indistinguishable change was observed in *E. coli* biofilm formation in the presence of *C. albicans* cells (Figure S1C). On the other hand, the fungal culture filtrate had no perceptible effect on the biofilm formation of either of the bacterial strains (Figure S1A–F). Interestingly, the bacterial strains failed to cater to any impact on each other’s biofilms (Figure S1A–D). To examine the biofilm composition in the presence and absence of the bacterial culture filtrates, a biofilm detachment assay was carried out by treating the preformed biofilms with sodium metaperiodate (NaIO_4_), proteinase K, and DNase I, which target the carbohydrate, protein, and nucleic acid components of the matrix, respectively (Figure 3D). As was reported earlier, we detected that proteins were the major components in all three entities, while carbohydrates and nucleic acids were ranked second and third [25,26]. In both the coculture conditions, we found a similar pattern of biofilm composition irrespective of the differential effect of the bacteria on candidal growth and biofilm development.

### 2.3. Chemical Characterization of the E. coli Filtrate Affecting the Growth and Biofilm of C. albicans

**Figure 4:**
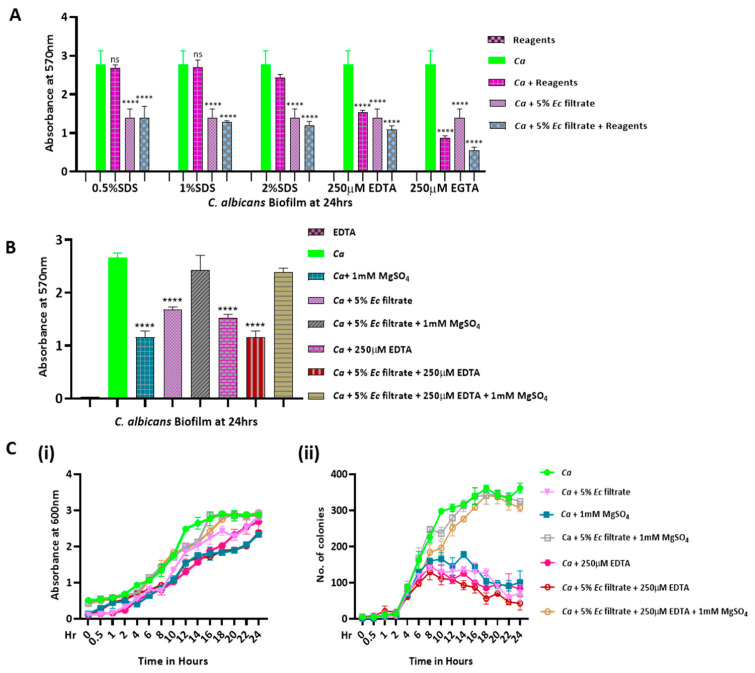
Chemical characterization of bacterial filtrate. (**A**) Preformed *Candida* biofilm was treated with *Ec* culture filtrate supplemented with indicated concentrations of reagents (SDS, EDTA, and EGTA) on a 24-well polystyrene plate for 24 h, and their absorbance was measured after staining with crystal violet. (**B**) Preformed *Candida* biofilm was treated with *Ec* culture filtrate supplemented with 250 μM EDTA and/or 1 mM MgSO_4_ as mentioned for 24 h on a 24-well polystyrene plate, and their absorbance was measured upon crystal violet staining. (**C**) For the growth curve and CFU assays, *Ca* culture, in the presence and absence of 5% of *Ec* culture filtrate, was supplemented with 1 mM MgSO_4_ and 250 µM EDTA as well as their combinations, and absorbance at OD_600nm_ (**i**) and CFU/mL (**ii**) were estimated. Mean values from three independent experiments are shown, and error bars represent the SEM. Graphs were plotted using GraphPad Prism version 8. **** indicates *p*-value < 0.0001 as significant, and *p*-value > 0.05 as non-significant (ns).

Next, to understand the chemical nature of the bacterial culture filtrate that affected fungal growth and biofilm development, we treated the filtrate with the denaturing agent SDS and the metal chelators EDTA and EGTA and then added it to the preformed *C. albicans* biofilm. We found that the SDS treated bacterial culture filtrate and SDS alone failed to deliver any significant change to the biofilm level of *C. albicans*, whereas both the metal chelating agents caused about a 50% reduction in the biofilm biomass irrespective of the presence or absence of the filtrate (Figure 4A). Moreover, EDTA and EGTA had an additive inhibitory effect when the *E. coli* culture filtrate was added to the *Candida* biofilm. Thus, we inferred that the *E. coli* culture filtrate functionally mimicked the metal chelators. To ascertain the chelating ability of the *E. coli* culture filtrate, a chemical complementation assay was carried out by supplementing it with MgSO_4_. We found a 1mM concentration of MgSO_4_ to be inhibitory to the candidal biofilm (Figure 4B). These reagents, SDS, EDTA, EGTA, and MgSO_4_, by themselves did not develop any coloration upon crystal violet staining. Interestingly, we noticed that MgSO_4_ rescued the candidal biofilm despite its own inhibitory action in the presence of the *E. coli* filtrate and EDTA. It suggested that a threshold level of magnesium is only required for fungal growth and that excess magnesium could be cytotoxic. To strengthen our results, growth curve and CFU analyses were carried out without and with the addition of a chelating agent or MgSO_4_, and, similar to the effect on biofilm formation, the growth as well as the CFUs of *C. albicans* were inhibited by the addition of 250 μM EDTA or 1 mM MgSO_4_; however, the inhibitory effect was reverted upon a 1 mM MgSO_4_ addition to the *E. coli* culture filtrate and EDTA (Figure 4(Ci,Cii)). These results suggested that the *E. coli* culture filtrate and/or the divalent metal chelating agents could function as a quorum-sensing molecule that might titrate out essential divalent metals, such as magnesium, to regulate *C. albicans* growth and biofilm formation.

### 2.4. Effect of S. aureus and E. coli Bacteria on the Growth and Biofilm of C. albicans in the Triple-Culture Condition

**Figure 5:**
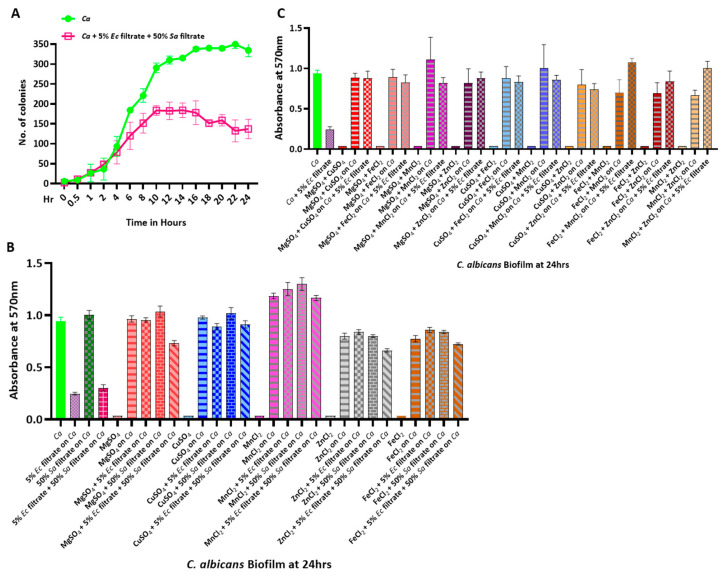
Growth and influence of metal ions on combinational trios of *C. albicans, S. aureus,* and *E. coli.* (**A**) CFU analyses of *Ca* culture after mixing with both of the bacterial culture filtrates (5% *Ec* and 50% *Sa*) on YPD + CHL + AMP media. (**B**) Preformed *Ca* biofilm was treated with bacterial culture filtrates containing 8 µM concentrations of divalent metal salts (MgSO_4_, CuSO_4_, MnCl_2_, ZnCl_2_, and FeCl_2_) for 24 h on a 48-well polystyrene plate and was further developed through crystal violet staining. (**C**) Preformed *Ca* biofilm was treated with *Ec* filtrate containing 8 µM concentrations of divalent metal salts in various combinations, such as MgSO_4_ + CuSO_4_, MgSO_4_ + FeCl_2_, MgSO_4_ + MnCl_2_, MgSO_4_ + ZnCl_2_, CuSO_4_ + FeCl_2_, CuSO_4_ + MnCl_2_, CuSO_4_ + ZnCl_2_, FeCl_2_ + MnCl_2_, FeCl_2_ + ZnCl_2_, and MnCl_2_ + ZnCl_2_, for 24 h and was further developed through crystal violet staining on a 48-well polystyrene plate. Salts without *Ca* biofilm were also used as controls. Mean values from three independent experiments are shown, and error bars represent the SEM.

The human gut harbors several bacterial species; therefore, we intended to determine the fungal growth in a polymicrobial situation by involving all three species in a single culture. We argued that, as MgSO_4_ rescued the loss of metal ions due to chelation by the *E. coli* culture filtrate, *S. aureus* could negate the inhibitory effect of the *E. coli* culture filtrate. To validate this, firstly, we determined the CFUs of *C. albicans* in a triple-culture condition, and, secondly, we determined the effect on the mature *C. albicans* biofilm of the mix of both the bacterial filtrates with and without supplementing various divalent metal compounds (Figure 5). The CFU analyses revealed that the addition of 5% of the *E. coli* filtrate combined with 50% of the *S. aureus* filtrate drastically reduced the *C. albicans* colony counts compared to that of the fungal monoculture (Figure 5A). This result suggested that the *E. coli* culture filtrate dominated the *S. aureus* filtrate and decreased the *C. albicans* CFU count, and this may be the case in the human gut as well. We also found a similar trend in *C. albicans* biofilm formation (Figure 5B). Furthermore, to check the specificity of the divalent metal ions affecting biofilm formation, a range of different divalent metal salts were tested. Since 1 mM MgSO_4_ was inhibitory to candidal growth, we determined the nontoxic minimal concentration of the compound and selected the 8 μM concentration for the subsequent assays (Figure S2). While the divalent salts, such as CuSO_4_, MnCl_2_, ZnCl_2,_ and FeCl_2_, alone did not affect biofilm maturation, similar to MgSO_4_ in the *Candida* monoculture, all of these divalent metals were able to compensate for the loss of cofactors in the triple-culture condition (Figure 5B). Next, we tried to estimate biofilm formation in a random combination of divalent metal ions with the *E. coli* culture filtrate (Figure 5C). Not only did various metal combinations suppress the inhibitory effect of the *E. coli* filtrate, many of them, such as FeCl_2_ + MnCl_2_, FeCl_2_ + ZnCl_2_, and MnCl_2_ + ZnCl_2_, also enhanced biomass formation significantly compared to that of the monoculture biofilm. This result suggested that divalent metals play very important roles in *C. albicans* growth and biofilm formation and that these metals are probably released from bacteria such as *S. aureus* in certain forms but are titrated out by EDTA-like reagents released from the bacterial cultures, such as that of *E. coli*, which could inhibit fungal growth and block the commensal-to-pathogenic transition of *C. albicans* in the host.

### 2.5. Effect of Polymicrobial Interaction on the Growth and Biofilm of Filamentation-Defective Strains of C. albicans

**Figure 6:**
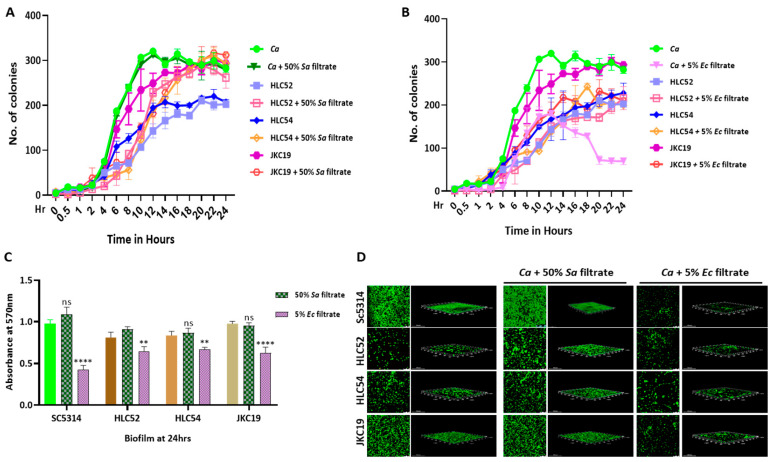
Growth and biofilm formation of filamentation-defective *C. albicans* strains in coculture conditions. CFU analyses of various *C. albicans* strains (WT, HLC52, HLC54, and JKC19) in the absence and presence of 50% of *Sa* (**A**) and 5% of *Ec* bacterial culture filtrates **(B)** in YPD media with selective antibiotics. (**C**) Preformed biofilms of above-mentioned strains were subjected to bacterial culture filtrates on a 48-well polystyrene plate, and retained biofilm was estimated using crystal violet staining followed by absorbance being recorded at 570 nm. Statistical analysis was performed using one-way ANOVA: ** *p*-value <0.01 and **** *p*-value < 0.0001 were significant, and *p*-value > 0.05 was nonsignificant (ns). (**D**) Biofilms of defective mutants under the bacterial filtrate treatments were imaged with a confocal scanning microscope.

To further strengthen our finding that the polymicrobial interaction is a key player in controlling *C. albicans* growth, we used three available mutant strains of *C. albicans* that showed different degrees of filamentation defects. While the double deletion of the transcription factors *EFG1* and *CPH1* (*efg1*ΔΔ*/cph1*ΔΔ) renders a complete loss in the morphological transition, their single homozygous deletions are partially or not defective in filamentation [27]. Therefore, we examined the effect of the bacterial culture filtrates on the growth and biofilm development of HLC52 (*efg1*ΔΔ), HLC54 (*efg1*ΔΔ*/cph1*ΔΔ), and JKC19 (*cph1*ΔΔ) *C. albicans* cells after verifying their filamentation status in the presence of serum (Figure S3). Surprisingly, the bacterial filtrates had a wide range of effects on the CFUs of these mutant fungal strains. While JKC19 behaved very similarly to the wild-type *C. albicans* strain and while its growth was suppressed in the presence of the *E. coli* culture filtrate, the growth of the other two mutants, HLC52 and HLC54, was not affected (Figure 6B). Interestingly, the enhancement of the growth of the HLC52 and HLC54 strains was quite apparent with the addition of 50% of the *S. aureus* culture filtrate (Figure 6A). However, the biofilm production of these strains did not change much in the presence of the *S. aureus* filtrate. While the *E. coli* culture filtrate significantly reduced the biofilm production of JKC19, it had a mild effect on the biofilm production of HLC52 and HLC54 (Figure 6C,D). Moreover, we observed that HLC52 and HLC54 exhibited lower CFU rates and poorer biofilm formation than those of the wild-type and JKC19 *C. albicans* strains under normal physiological conditions.

### 2.6. E. coli Cell-Free Culture Filtrate Suppressed Virulence Gene Expression of C. albicans and Systemic Candidiasis Development in Mice

**Figure 7:**
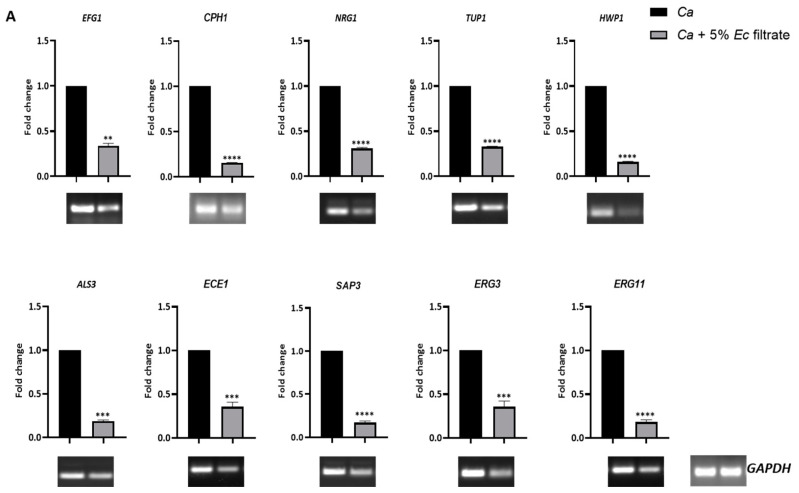

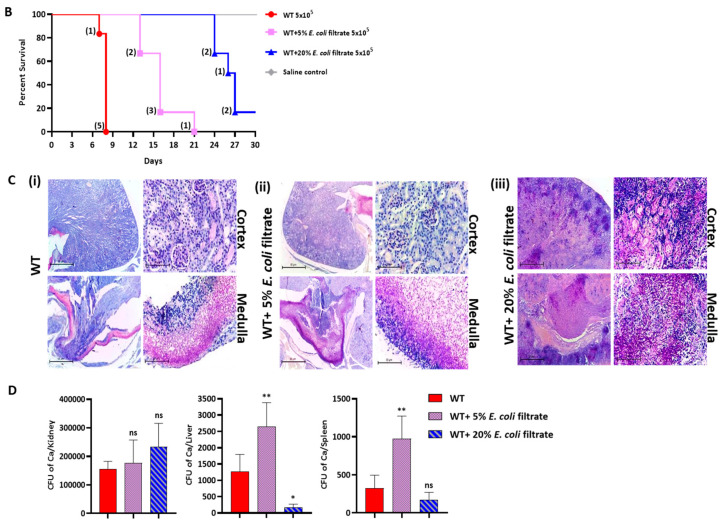
Virulence gene expression and pathogenesis of *C. albicans*. (**A**) Total RNA was isolated from *C. albicans* cells without and with treatment with 5% *Ec* culture filtrate for 6 h, and cDNA was synthesized. Expression of virulence genes, such as *EFG1*, *CPH1*, *NRG1*, *TUP1*, *HWP1*, *ALS3*, *SAP3*, and *ECE1*, was compared through real-time and semiquantitative PCR. The data are mean values from three independent experiments, and error bars represent the SEM. (**B**) BALB/c mice (*n* = 6/category) were injected intravenously with 5 × 10^5^ cells of *C. albicans* (WT), WT + 5% of *Ec* filtrates, and WT + 20% of *Ec* filtrates, and their survivability was monitored. The number of mice that were dead on a particular day was mentioned. (**C**) PAS-stained kidney sections show the fungal burden in the medulla and cortex. (**D**) CFU analyses of harvested organs, such as kidney (**i**), liver (**ii**), and spleen (**iii**), are shown. Fungal population determination was carried out on YPD agar plates supplemented with CHL (100 µg/mL). This analysis involved three independent experiments. The survival curve and CFU analyses were plotted using GraphPad Prism Software version 8.0. Statistical analysis was performed using one-way ANOVA: * *p*-value < 0.05, ** *p*-value < 0.01, *** *p*-value < 0.001, and **** *p*-value < 0.0001 were significant; *p*-value > 0.05 was nonsignificant (ns).

The morphological switching of *C. albicans* is also associated with altered virulence gene expression. When the *C. albicans* cell switches its morphology from the yeast form to pseudohyphal structures to hyphal structures, the expression of virulence-associated genes increases. Since the *E. coli* cell-free filtrate affected the growth, biofilm formation, and morphology of *C. albicans*, we determined whether this polymicrobial interaction also affected the virulence gene expression by using quantitative real-time (qRT) and semiquantitative end-point-PCR analyses (Figure 7A). The total RNA of the 5% *E. coli* filtrate-treated *C. albicans* was isolated, and the cDNA expression of the genes involved in morphogenesis (*EFG1*, *CPH1*, *NRG1*, *TUP1*, and *HWP1*) and the genes involved in adhesion and penetration (*ALS3*, *ECE1*, *SAP3*, *ERG3*, and *ERG11*) was determined [28,29,30]. In the planktonic interaction, the expression of all of these genes was mostly reduced, indicating that *E. coli* can affect the pathogenesis of *C. albicans*.

The intravenous challenge of *C. albicans* in mice causes systemic candidiasis, and animals succumb due to essential organ failure, a condition that mimics human systemic candidiasis [31]. To determine the pathogenicity, BALB/c mice (*n* = 6) were injected with untreated and *E. coli* cell-free-filtrate-treated *C. albicans* cells. Both treated and untreated *C. albicans* cells were thoroughly washed and diluted in PBS to prepare the inoculum for the animal study. An inoculum size of 5 × 10^5^ CFUs of *C. albicans* per mouse (100 μL) was injected via the lateral tail vein, and the longevity of the mice with the fungal challenge was monitored (Figure 7B). Similarly, for the control experiment, an equal volume of PBS was injected. The mice that suffered due to severe candidiasis succumbed or were sacrificed based on the characteristics of humane endpoints. While most of the mice challenged with untreated *C. albicans* succumbed to the infection within 8 days of inoculation, there was a significant improvement in the survivability of the mice group injected with 5% *E. coli* culture-filtrate-treated *C. albicans*, and the survival duration improved to a median of 15 days. Furthermore, to find out the concentration-dependent effect of the *E. coli* culture filtrate on the fungal pathogenesis, mice were challenged with 20% *E. coli* culture-filtrate-treated *C. albicans* cells. Interestingly, 20% of the mice challenged with the higher-dosage *E. coli* culture-filtrate-treated *C. albicans* cells did not succumb to infections, while the others died with an improved median survival duration of 25 days. These results suggested that the *E. coli* culture filtrate attenuates the virulence of *C. albicans* in a dose-dependent manner (Figure 7B). Irrespective of the type of inoculum, the histopathology of the PAS-stained kidney sections showed the presence of *C. albicans* cells in the kidneys of the dead mice (Figure 7C). The medulla of the kidney possessed more fungal cells than the cortex. However, the delayed-death mice showed a fungal load in the cortex as well. The CFU analysis of the vital organs, such as the kidney, liver, and spleen, also confirmed the death of the mice being due to the presence of *C. albicans* cells (Figure 7D). The fungal load in the kidney was very high (~1.5 × 10^5^ cells) in comparison to that in the liver and spleen (~250–2500 cells). More importantly, we observed a higher fungal burden in the CFUs and PAS-stained kidneys of the mice inoculated with the 20% *E. coli* culture filtrate treated as compared to wild-type and the 5% *E. coli* filtrate-treated *C. albicans*. This also suggested that a higher fungal accumulation of the 20% *E. coli* culture-filtrate-treated *C. albicans* cells in the kidney is required to cause systemic candidiasis. Altogether, our results suggested that *E. coli* can attenuate the virulence of *C. albicans* by suppressing virulence gene expression, growth, biofilm formation, and the morphological transition.

## 3. Discussion

**Figure 8:**
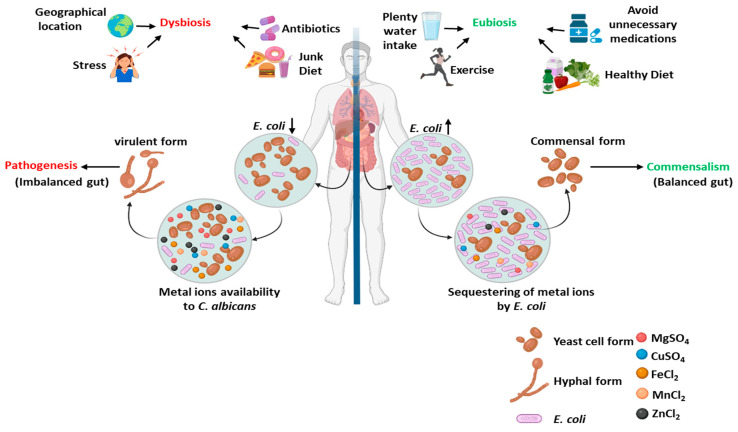
Nutritional immunity with respect to *E. coli***.** A working model depicting the importance of *E. coli* in suppressing the growth and pathogenesis of *C. albicans*. Microbial homeostasis and the host’s immunity maintain *C. albicans* in a commensal state, and *E. coli* plays a predominant role by helping the host in maintaining nutritional immunity. Due to dysbiosis, *E. coli* or other such good bacteria populations decrease, causing the sufficient availability of metals and nutrients for promoting *C. albicans* growth and infections.

About 40–70% of healthy individuals carry *C. albicans* in their guts, where it survives asymptomatically. Since humans are not immune to *C. albicans* infections, there may be underlying mechanisms in the host that suppress the pathogenic traits of this opportunistic fungus; therefore, the fungus maintains a long-term mutualism with the host [4,6]. One assumption is that, as humans are colonized by a full array of microorganisms irrespective of either being in healthy or diseased states, neighboring microbes could influence the pathogenesis of *C. albicans* [32,33]. Polymicrobial interactions could have either synergistic or antagonistic impacts on the commensal or pathogenic outcome of *C. albicans*. Several bacterial species, especially *E. coli*, *P. aeruginosa*, *Helicobacter pylori*, *Lactobacillus* spp., *Mycobacterium tuberculosis*, *Salmonella* spp., *Streptococcus* spp., *Staphylococcus* spp., and fungal species mostly from the genus *Candida*, have been coisolated from various human organs [34,35,36]. Our 16S and 18S rRNA amplicon sequencing of the fecal meta-DNA of mice also suggested the existence of a similar community in animals [4]. Chronic infections are found to be mostly polymicrobial. *S. aureus* often coinfects with species such as *Haemophilus influenza*, *Enterococcus faecalis*, *P. aeruginosa*, *Streptococcus pneumoniae*, *Corynebacterium* spp., *Lactobacillus* spp., *C. albicans*, and even the influenza virus. Chronic wounds are a reservoir of several fungal species from the *Candida*, *Curvularia*, *Aureobasidium*, *Cladosporium*, *Ulocladium*, *Engodontium*, *Malassezia*, and *Trichophyton* genera, albeit *Candida* species are predominant [35,37]. Since *E. coli* and *S. aureus* survive as natural neighbors of *C. albicans* both in the healthy and disease states of humans, this study was planned to decipher the impact of polymicrobial interactions on the growth and pathogenesis of *C. albicans.* Our study revealed that live *E. coli*, but not *S. aureus*, cells suppress the growth, filamentation, and biofilm development of *C. albicans.* Even the cell-free culture filtrate of *E. coli* had a similar influence. Moreover, the *E. coli* culture filtrate decreased the virulence gene expression of *C. albicans*, and the animals infected with *E. coli* culture-filtrate-treated *C. albicans* survived longer than the untreated *C. albicans* infected mice group. This suggested that *E. coli* plays a very important role in regulating the pathogenic trait of *C. albicans* and that it could block the commensal-to-pathogenic transition of *C. albicans* in the host’s gut (Figure 8). Alternatively, another study reported that only β-lactam antibiotics induce a peptidoglycan storm by killing the bacteria causing the hyphal transition and promoting the invasive growth of *C. albicans* [10]. Interestingly, our triple-culture system further confirmed the dominant role of *E. coli*. In our study, neither the bacteria nor *C. albicans* influenced the growth and biofilm of the bacterial species. More importantly, the inhibitory effect of *E. coli* on the strain of *C. albicans* that underwent the hyphal transition (WT and JKC19) and of neutrality on the filamentation-defective avirulent strains (HLC52 and HLC54) suggested that *E. coli* can differentiate among the fungal strains and selectively suppress the growth of only the virulent strain. In an effort to characterize the chemical nature of the *E. coli* filtrate, this study found that the soluble compound(s) released from the growing *E. coli* cells functionally mimics the metal chelators. This was further authenticated by the chemical complementation of the divalent metals. We found that the supplementation of compounds such as MgSO_4_, CuSO_4_, FeCl_2_, MnCl_2_, and ZnCl_2_ rescued the biofilm formation ability that was inhibited in the presence of *E. coli* and EDTA. Thus, we concluded that *E. coli* by releasing soluble factors analogues to EDTA sequesters the essential metals away from the growing environment or that released by bacteria like *S. aureus* to suppress the pathogenic attributes of *C. albicans.* Thus, it serves as a checkpoint of pathogenic transition from a commensal state of *C. albicans*. 

This study also demonstrated the importance of divalent metals in the growth and biofilm development of *C. albicans.* Several reports have already demonstrated the essentiality of metals and various modes of uptake as well as their utilization in fungi, including *C. albicans*. Metals play a central role in *C. albicans* infection processes, as they serve as cofactors in several proteins involved directly and indirectly in virulence [38,39,40]. Iron is mostly required for the tricarboxylic acid (TCA) cycle and the electron transport chain via its direct incorporation or by acting as the prosthetic group in the active sites of essential enzymes. Most bacteria and fungi secrete a heterogeneous class of small molecules called siderophores for extracellular ferric iron uptake; however, a few fungi, such as *C. albicans*, do not secrete siderophores and rather rely on xenosiderophores [41]. *SIT1* encodes a dedicated xenosiderophore transporter in *C. albicans* which is required for the invasion of human epithelial cells in vitro, although its deletion did not result in the attenuation of virulence [42]. In addition, *C. albicans* can directly or indirectly obtain iron from the host’s hemoglobin, hemin, ferritin, and transferrin [43]. *C. albicans* takes up ferritin by bindings to Als3, which is also a protein required for cell adhesion and invasion. The *C. albicans* strain lacking Als3 did not bind ferritin, grew poorly in the presence of ferritin, and was unable to damage epithelial cells [44]. Moreover, our study also found a lowered expression of *ALS3* when the fungal cells were treated with the *E. coli* filtrate. Thus, in addition to metal sequestration, *E. coli* can suppress the expression of certain metal receptors in *C. albicans* to affect fungal growth. Similar to iron, zinc is also a structural and catalytic cofactor for several proteins involved in endoplasmic reticulum (ER) functioning, oxidative stress resistance, protein folding, vesicular trafficking, and chromatin modification [45]. SODs, essential enzymes for the detoxification of reactive oxygen species, are copper-, manganese-, and zinc-dependent enzymes [46]. Fungi express zinc importers and uptakers, known as zincophores, to obtain zinc from the host environment [47]. *C. albicans* employs Zrt1 and Zrt2 as eukaryotic zinc transporters: ZRT-IRT-like proteins (ZIPs) and zinc uptake increase in the early stages of *C. albicans* infections in mice [48]. A recent study suggested that Sap6, a secreted aspartic proteinase, is also involved in zinc uptake [49]. Copper is another essential trace element required for many biochemical reactions. While the mitochondrial cytochrome C oxidase requires Cu for its function in the electron transport chain, cytoplasmic or cell-wall-associated Cu/Zn- and Mn-SODs can protect fungal cells from externally and internally generated oxidative stress [50]. *C. albicans* copper transporters are upregulated upon phagocytosis [51]. Manganese is required for the functioning of polymerases, the sugar transferases of the Golgi apparatus, and mitochondrial Mn-SODs [52]. External manganese is taken up by Smf1 and Smf2 transporters; upon internalization, it can then be transported by the Golgi P-type Ca^2+/^Mn^2+^ ATPase, Pmr1 [53]. Pmr1 is required for *C. albicans* virulence [54]. Magnesium is another essential micronutrient involved in cell signaling, ATP synthesis, and nucleic acid metabolism. A recent report suggested that a lack of Mg affects the drug resistance mechanisms and immune evasion of *C*. *albicans* [55].

Since divalent metals are also equally essential, to counteract metal uptake by microorganisms, hosts are equipped with a strategy called “nutritional immunity” by which they limit the availability of metals for the invading pathogens [39,40]. For example, the antimicrobial peptides, histatins, in saliva can sabotage zinc and copper ions [56]. Immune cells, such as T cells, macrophages, and dendritic cells, alter the expression of zinc transporters, such as ZIP8, zinc exporters, and zinc importers, to decrease the cellular zinc concentration [57,58]. The host protein calprotectin inhibits fungal growth by chelating most of the transition metals [59,60]. Similarly, the bovine pancreatic trypsin inhibitor (BPTI) and the metal chelator diethylenetriamine pentaacetic acid (DTPA) inhibit the growth of *C. albicans* by sequestering cellular Mg [61,62]. In this study, we found that the *E. coli* cell-free culture filtrate alone could reduce the availability of all these metals and could add to the nutritional immunity of the host. Taken altogether, our study found a novel role of *E. coli*, a neighboring bacteria of *C. albicans* in most human organs: it functionally mimics a chelating agent to control the fungal pathogenesis. It might also help the host enhance its nutritional immunity. Further study is required to determine the underlying processes behind the *E. coli–C. albicans* interaction, which could provide critical information for the prevention and treatment of *C. albicans* infections.

## 4. Materials and Methods

### 4.1. Animal Ethical Clearance

Mice and related experimental protocols were approved by the Institutional Animal Ethical Committee, Institute of Life Sciences, Bhubaneswar, India, with permit number ILS/IAEC-133-AH/AUG-18. All the mice experiments were conducted in strict accordance with the guidelines of the institute. 

### 4.2. Animals, Reagents, Strains, and Growth Conditions

BALB/c female mice of 5–7 weeks old were procured from the institute animal house and were maintained in individually ventilated cages under standard ad libitum conditions. Oligonucleotides used in RT-PCR were procured either from Eurofins scientific or Integrated DNA Technologies. Periodic Acid Schiff (PAS) Stain Kit from Abcam, Cambridge, MA, USA, was obtained. *C. albicans* (SC5314/WT), HLC52 (defective in *efg1*), HLC54 (defective in both *cph1* and *efg1*), and JKC19 (defective in *cph1*) strains were cultured in liquid YPD growth media (1% yeast extract, 2% peptone, and 2% glucose in distilled water), whereas the two bacterial strains, *S. aureus* (ATCC43300) and *E. coli* (MG1655), were cultured as a monoculture in BHI (7.7 g of calf brain, 9.8 g of beef heart, 10 g of protease peptone, 2 g of dextrose, 5 g of sodium chloride, and 2.5 g of disodium phosphate per liter) and LB (10 g of tryptone, 5 g of yeast extract, and 10 g of NaCl per liter) nutrient media, respectively. For coculture experiments, YPD was used. 

### 4.3. Extraction of Bacterial Filtrate 

*S. aureus* and *E. coli* precultures were grown in their respective nutrient media at 37 °C for 16 h with orbital shaking at 200 RPM. The cultures were subsequently diluted in fresh YPD to an OD_600nm_ of 0.01 and were allowed to grow to an OD_600nm_ of 0.1 (1 × 10^7^ cells/mL). The obtained culture was centrifuged at 6000 RCF for 15 min. The culture was further filter sterilized using a 0.2-micron syringe filter to obtain a cell-debris-free filtrate. The processes of filtration and storage were collectively carried out in sterile conditions. Freshly prepared culture filtrates were used in each experiment if required.

### 4.4. Growth Curve and CFU/mL Analysis

For the growth curve and CFU analyses, we used a protocol as described previously [23]. Briefly, for the coculture experiment, equal volumes of *C. albicans* (OD_600nm_ of 0.5) and the bacterial culture (OD_600nm_ of 0.1) in 20 mL of YPD medium were mixed and allowed to grow at 37 °C with shaking at 200 RPM. The absorbance was measured at various time points to generate growth kinetics. Similarly, monoculture growth curves were also generated. For survival assays, the coculture mix was serially diluted in autoclaved water, and the dilutions (approx. 50 µL) were spread onto the agar plates supplemented with antibiotics. Amphotericin-B-supplemented (100 µg/mL) BHI agar plates were used to quantify *S. aureus*, and chloramphenicol-containing (100 µg/mL) YPD agar plates were used to detect *C. albicans* for the *C. albicans*–*S. aureus* coculture experiment. Similarly, the serial dilutions of *C. albicans–E. coli* cocultures were plated on YPD and LB agar plates treated with ampicillin (100 µg/mL) and amphotericin B (100 µg/mL) to quantify *C. albicans* and *E. coli*, respectively. *C. albicans* culture with 5% of bacterial culture filtrates was also assessed in the same way as that of pure cultures. The effect of filtrates was also examined by adding them after *C. albicans* reached its mid-log phase during the 8th h. In our next CFU assay, *S. aureus*–*C. albicans* (1:1 ratio) culture mix was directly treated with 4–8 µg/mL concentrations of chloramphenicol (CHL), and, similarly, 2.5–5 µg/mL concentrations of ampicillin (AMP) were added to *E. coli*–*C. albicans* culture mixtures. The CFU/mL was measured for each situation, and a graph of these measurements was plotted against time using GraphPad Prism version 8.0.

### 4.5. Biofilm Growth and Confocal Laser Scanning Microscopy (CLSM)

Biofilms of *C. albicans* and bacterial strains were developed by growing diluted overnight culture in YPD broth (OD_600nm_ = 0.5) on a 24-, 48-, or 96-well polystyrene plate, depending upon the number of samples, for 24 h in static conditions as described before [19,21]. Biofilm development of HLC52, HLC54, and JKC19 *C. albicans* strains was carried out at 30 °C, whereas that of WT *C. albicans* and bacterial cultures was performed at 37 °C. After 24 h of biofilm formation, about 200–500 μL of microbial pure-culture cells or their cell-free culture filtrates (5, 25, and 50%) were added and incubated again for another 24 h. To characterize the chemical nature of filtrates, biofilms were developed as above and were treated with either 5% of *Ec* filtrate or 50% of *Sa* filtrate alone or with mentioned concentrations of SDS/EDTA/EGTA/MgSO_4_/other metal compounds as well as with compounds alone. After a total of 48 h of incubation, the supernatant was gently removed without disturbing the biofilm, and the wells were washed with 1X PBS twice. The biofilm obtained was treated with 0.1% crystal violet and was left for 20 min to stain at 25 °C. The plate was allowed to air dry, and the bound dye was then resuspended in 33% glacial acetic acid. Then, absorbance was recorded at 570 nm. The experiments were repeated twice with biological triplicates. Similarly, biofilms were formed and treated on 8-well chamber slides; images were obtained after staining with 1% acridine orange using Leica TCS SP8 confocal scanning system, and excitation at 483 nm and emission with 500 to 510 nm band-pass filter were used. 

### 4.6. Biofilm Detachment Assay

Biofilms of *C. albicans* were developed on 24-well polystyrene plates as described above. The mature biofilms that were cultivated were treated with 500 μL of DNase I (0.5 mg/mL), proteinase K (0.1 mg/mL), and NaIO_4_ (40 mM) in their assigned wells and were allowed to incubate for 24 h more in static conditions at 37 °C. Following the treatment, the biofilm obtained was assessed using crystal violet staining. The experiment was repeated thrice.

### 4.7. RNA Isolation, Real-Time PCR, and qRT-PCR

The fungal culture without and with 5% of *E. coli* cell-free culture filtrate was cultivated in YPD medium after 6 h of growth at 37 °C in 200 RPM shaking conditions. The total RNA was isolated using the MagSure All-RNA Isolation Kit, and cDNA was synthesized from 1000 ng of RNA using a High-Capacity cDNA Synthesis Kit (Thermo Fischer Scientific, Waltham, MA, USA). The cDNA stock was diluted with nuclease-free water (1:29) to prepare the working concentration. The cDNA was used as a template in RT-PCR along with 10 pmoles of primers and 2x SYBR Green reagent (Applied Biosystems™ PowerUp™ SYBR™ Green Master Mix, Thermo Fischer Scientific). The amplification program consisted of the cyclic condition, which comprises 95 °C for 10 min during the holding stage; 95 °C for 15 s and 60 °C for 1 min during the PCR-cyclic stage; and 95 °C for 15 s, 60 °C for 1 min, and 95 °C for 15 s during melting-curve stage, and there was a 4 °C hold per cycle. Semiquantitative RT-PCR assay was preceded with 10X PCR buffer, 10 mM dNTPs, 10 pmoles of primers, and DNA Taq polymerase. Samples were checked on an agarose gel. All the PCR primers and cyclic conditions are mentioned in Table S1. 

### 4.8. Animal Studies

The mice (*n* = 6) from each category were intravenously injected with 5 × 10^5^ CFU/mL of *C. albicans* (WT), WT + 5% of *E. coli* culture filtrate, WT + 20% of *E. coli* culture filtrate, and saline as a control. The inoculums were prepared from the freshly grown *C. albicans* culture without or with treatment with mentioned filtrates for 6 h at 37 °C. With the completion of incubation, the cultures were pelleted down at 10,000 RPM for 1 min and were resuspended in 1X PBS. The *Candida* cells were counted using a Neubauer chamber slide and online hemocytometer software. The survival of mice was monitored for 30 days. The organs (kidney, liver, and spleen) were dissected from the sacrificed mice. Amidst both the kidneys, one kidney was used for CFU determination, and another was used for histology through PAS staining and imaging under a Leica DM500 microscope. The organs were homogenized, and serial dilutions were plated on YPD solid agar plates supplemented with 100 μg of chloramphenicol antibiotic to determine CFU/organ. The CFU/organ and survival curve was plotted using GraphPad Prism 8 software. The in vivo study was repeated thrice.

## Data Availability

Not applicable.

## Supplementary Materials

The following supporting information can be downloaded at: https://www.mdpi.com/article/10.3390/jof9030286/s1, Figure S1: Bacterial biofilm estimation and CLSM, Figure S2: Quantification of Mg2+ ions, Figure S3: Morphology assessment of *C. albicans* filamentation-defective mutants in the presence of serum, and Table S1: Primers used during RT-PCR and Semi-quantitative analyses.

## Abbreviations

EDTA represents ethylene diamine tetraacetic acid; EGTA represents ethylene glycol-bis (2-amino ethyl ether)-N, N, N′, N′-tetraacetic acid; and SDS represents sodium dodecyl sulfate.
