# Supplementary material for: Escherichia coli, but Not Staphylococcus aureus, Functions as a Chelating Agent That Exhibits Antifungal Activity against the Pathogenic Yeast Candida albicans
Source: J Fungi (Basel). 2023 Feb 22;9(3):286. doi: 10.3390/jof9030286 (PMC10057578; doi:10.3390/jof9030286)
Supplement: Supplementary file 1 [file jof-09-00286-s001.zip › jof-2129046-supplementary.pdf]

Escherichia coli, but Not *Staphylococcus aureus*, Functions as a Chelating Agent that Exhibits Antifungal Activity against the Pathogenic Yeast *Candida albicans*

Swagata Bose [1,2], Durg Vijai Singh [3], Tapan Kumar Adhya [2] and Narottam Acharya[1,*]

[1]Department of Infectious Disease Biology, Institute of Life Sciences, Bhubaneswar-751023, India.

[2]KIIT School of Biotechnology, Bhubaneswar-751021, India.

[3]Department of Biotechnology, School of Earth, Biological and Environmental Sciences, Central University of South Bihar, Gaya- 824236, India.

*Correspondence to:

Narottam Acharya, Phone: 91-674-2304278, Fax: 91-674-230 0728

E-mail: narottam_acharya@ils.res.in; narottam74@gmail.com

Running title: Regulation of polymicrobial interaction

Keywords: Candidiasis, Morphogenesis, nutritional immunity, peptidoglycan, biofilm, pathogenesis, quorum sensing, divalent metals,

Figure S1 Bacterial biofilm estimation and CLSM. (A) Biofilm of *Sa* pure culture and co-culture with 5% of *Ca* and *Ec* culture filtrates; (B) quantification of *Sa* biofilm in the presence of 5%, 25%, and 50% of *Ca* and *Ec* culture filtrates. Similarly, (C) Biofilm of *Ec* pure culture and co-culture with 5% of *Ca* and *Sa* culture filtrates. (D) *Ec* biofilm estimation in the presence of 5%, 25%, and 50% of *Ca* and *Sa* filtrates. Graph plotted using Graph Pad Prism 8.0. (E, F) For CLSM, the bacterial biofilm matrices were grown in the 8-well chambered slides and visualized under 40x Leica TCS SP5 confocal scanning microscope. Mean values from three independent experiments are shown, and error bars represent the SEM. P-value <0.05; ****: P-value <0.0001 (significant) and ns: >0.05 (non-significant).

Figure S2 Quantification of Mg^{2+} ions. Threshold concentration of divalent ions estimated using different range of Mg^{2+} ion concentrations ranging between 62.5µM to 0.24µM on pre-formed *Ca* biofilm. The graph was plotted using Graph Pad Prism 8.0. Mean values from three independent experiments are shown, and error bars represent the SEM. P-value <0.05; ****: P-value <0.0001 (significant) and ns: >0.05 (non-significant).

Figure S3 Morphology assessment of *C. albicans* filamentation-defective mutants in the presence of serum. *C. albicans strains* (WT, HLC52, HLC54, and JKC19) were grown in YPD media without and with 10% FBS serum at 30°C with continuous shaking, the morphology imaged at 30mins (A), and at 3hrs time interval (B). The imaging was carried under 40x microscope (Leica DM500).

Table S1: Primers used during RT-PCR and Semi-quantitative analyses. Sequences of primers and cyclic conditions used for amplification of *C. albicans* genes used in this study were given.

Targets	Sequences (5'-3')	Cyclic conditions	Amplicon size (bp)
GAPDH	(Fp) GACCGTTGACGGTCCATCC<br>(Rp) CATCGGTGGTTGGGACTC	D at 95°C for 15″<br>A at 52°C for 30″<br>E at 72°C for 30" } 30cycles	161
ALS3	(Fp) CGGTTGCGACTGCAAAGA C<br>(Rp) GACCAACCCAAAACAGCATTCC	D at 95°C for 15″<br>A at 55°C for 30″<br>E at 72°C for 30" } 30cycles	127
HWP1	(Fp) CAGTTCCACTCATGCAACCATC<br>(Rp) GCAATACCAATAATAGCAGCACCG	D at 95°C for 15″<br>A at 55°C for 30″<br>E at 72°C for 30″ } 30cycles	115
ERG11	(Fp) TTACCTCATTATTGGAGACGTGATG<br>(Rp) CACGTTCTCTTCTCAGTTTAATTTCTTTC	D at 95°C for 15″<br>A at 52°C for 30″<br>E at 72°C for 30" } 30cycles	85
ERG3	(Fp) TCCAGTTGATGGGTTCTTCC<br>(Rp) GGACAGTGTGACAAGCGG	D at 95°C for 15″<br>A at 55°C for 30″<br>E at 72°C for 30″ } 30cycles	179
SAP3	(Fp) GTTACTGGTCCCCAAGGTG<br>(Rp) CTTGTCCTTGACCAGCTTGAC	D at 95°C for 15″<br>A at 55°C for 30″<br>E at 72°C for 30″ } 30cycles	208
EFG1	(Fp) CCAGGGTGGTGCTGCTAATAG<br>(Rp) GGGTGAAGGGTGAACTGAACC	D at 95°C for 15″<br>A at 55°C for 30″ } 30cycles	241

		E at 72°C for 30″	
CPH1	(Fp) GACGCTTCTGGGTTT CC<br>(Rp) ATCCCATGGCAATTTGTTG	D at 95°C for 15″<br>A at 54°C for 30″ } 30cycles<br>E at 72°C for 30″	160
NRG1	(Fp) CACCTCACTTGCAACCCC<br>(Rp) GCCCTGGAGATGGTCTGA	D at 95°C for 15″<br>A at 55°C for 30″ } 30cycles<br>E at 72°C for 30″	198
TUP1	(Fp) CTCTTGGCGACAGGTGCAG<br>(Rp) GTGGTGACGCCGTCTTCGA	D at 95°C for 15″<br>A at 55°C for 30″ } 30cycles<br>E at 72°C for 30″	224
ECE1	(Fp) GAGATGGCGTTCCAGATGTT<br>(Rp) CCGGCATCTCTTTTAACTGG	D at 95°C for 15″<br>A at 55°C for 30″ } 30cycles<br>E at 72°C for 30″	214

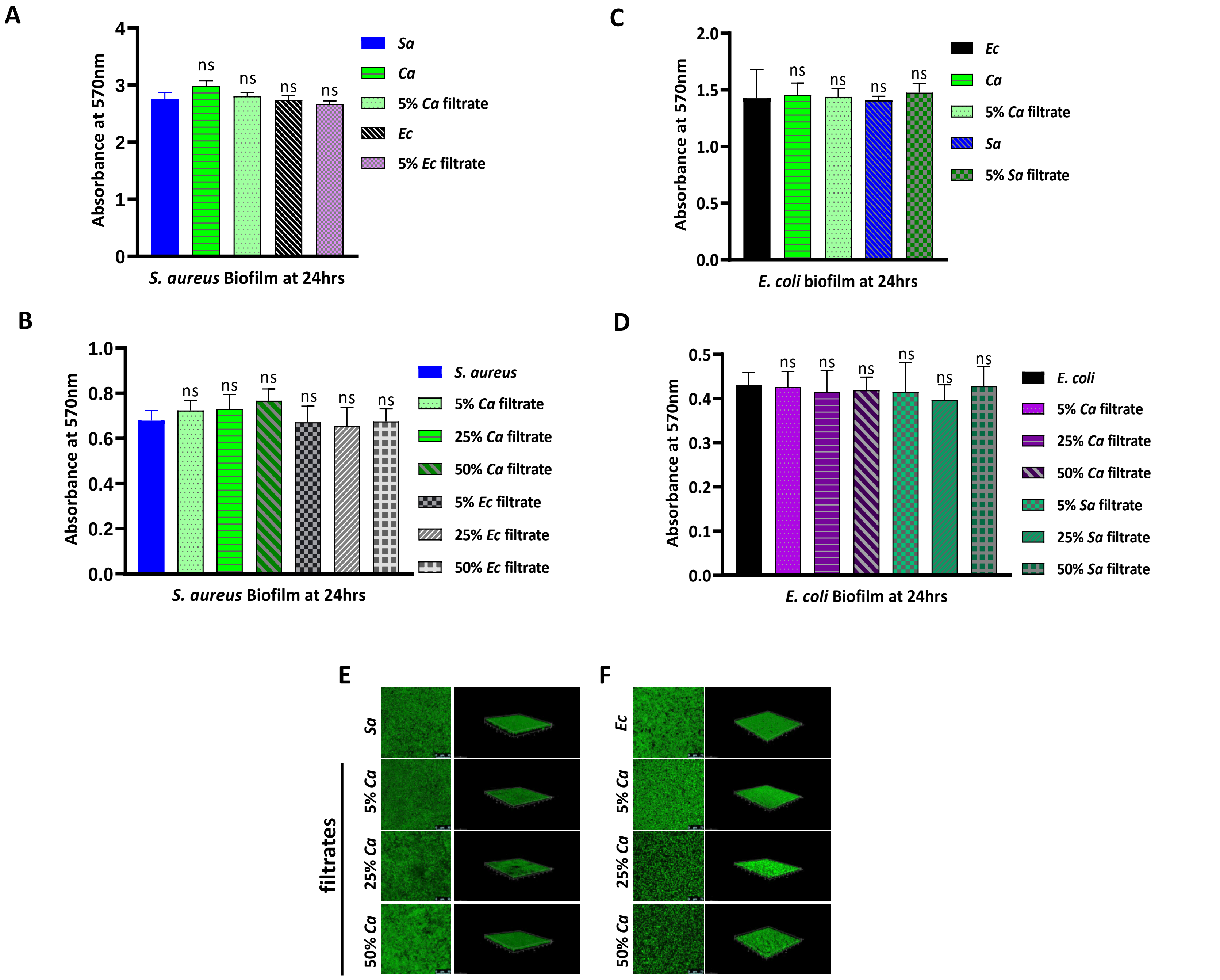
A
Absorbance at 570nm
ns
Sa
Ca
5% Ca filtrate
Ec
5% Ec filtrate
S. aureus Biofilm at 24hrs
C
E. coli biofilm at 24hrs
5% Sa filtrate
B
S. aureus
25% Ca filtrate
50% Ca filtrate
5% Ec filtrate
25% Ec filtrate
50% Ec filtrate
D
E. coli
25% Sa filtrate
50% Sa filtrate
E. coli Biofilm at 24hrs
E
F
filtrates
5% Ca
25% Ca
50% Ca

Figure S1

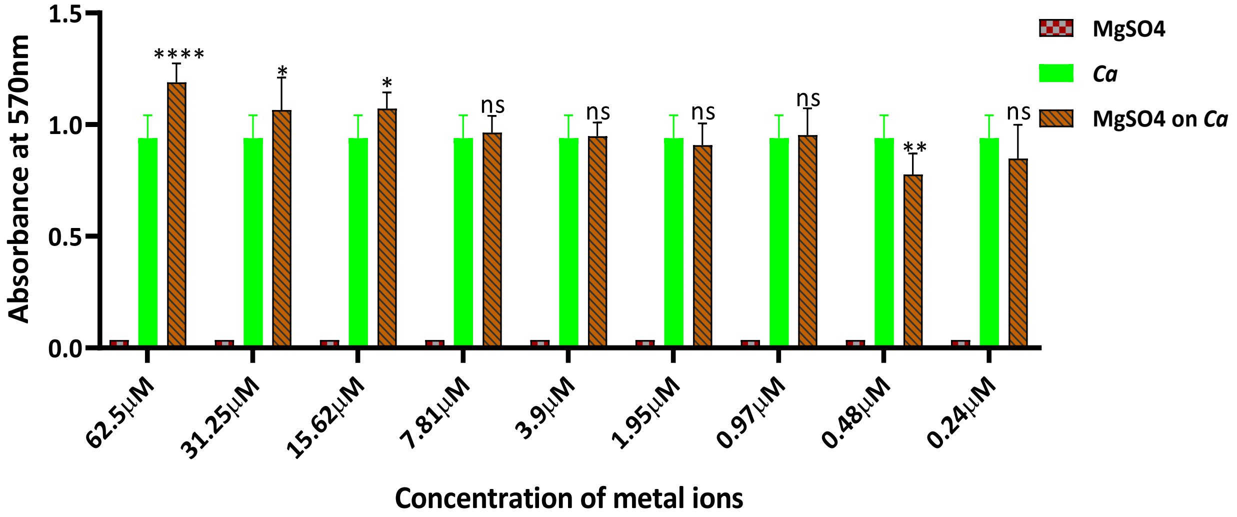

Absorbance at 570nm
1.5
1.0
0.5
0.0
****
*
*
ns
ns
ns
ns
**
ns
62.5µM
31.25µM
15.62µM
7.81µM
3.9µM
1.95µM
0.97µM
0.48µM
0.24µM
Concentration of metal ions
MgSO4
Ca
MgSO4 on Ca

Figure S2

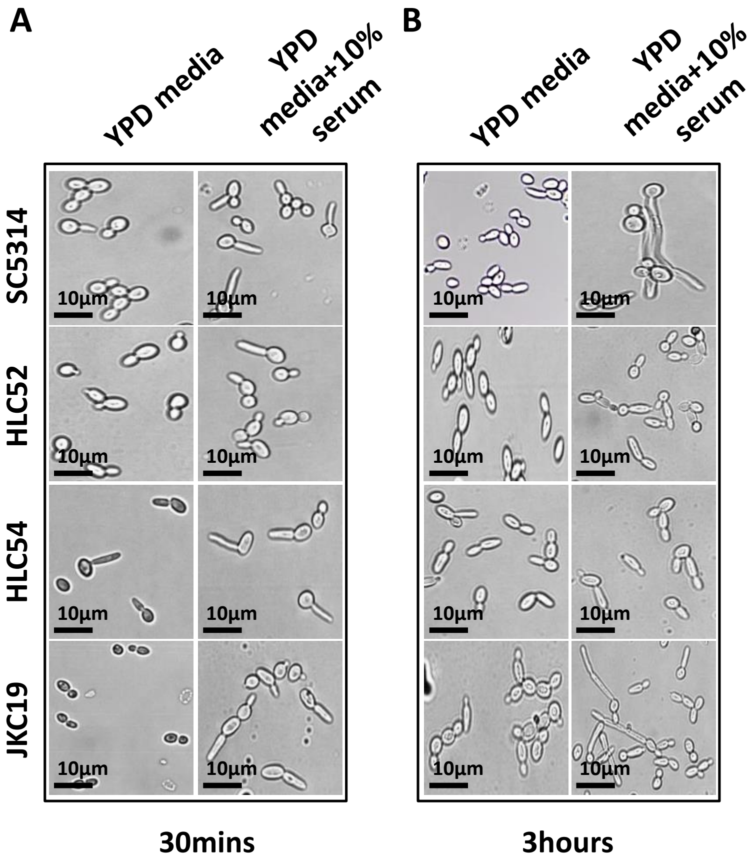
A
B
YPD media
YPD media+10% serum
YPD media
YPD media+10% serum
SC5314
HLC52
HLC54
JKC19
10µm
30mins
3hours

Figure S3
